# Arthroscopic Diagnosis and All‐Inside Repair of Meniscofibular Ligament Tear

**DOI:** 10.1002/atn2.70339

**Published:** 2026-10-23

**Authors:** Alberto Grassi, Tosca Cerasoli, Vito Gaetano Rinaldi, Claudio Rossi, Giulio Maria Marcheggiani Muccioli, Stefano Zaffagnini

**Affiliations:** ^1^ Department of Biomedical and Neuromotor Sciences (DIBINEM) Alma Mater Studiorum University of Bologna Bologna Italy; ^2^ 2nd Orthopaedic and Traumatologic Clinic IRCCS Rizzoli Orthopaedic Institute Bologna Italy

## Abstract

Meniscofibular ligament (MFL) tears are an under‐recognized source of lateral meniscal instability, frequently encountered in association with anterior cruciate ligament injury. Failure to identify and address MFL insufficiency may result in persistent posterolateral pain and meniscal hypermobility despite successful anterior cruciate ligament reconstruction. This Technical Note describes a reproducible, all‐inside arthroscopic technique for the diagnosis and repair of MFL tears. Diagnostic arthroscopy includes systematic evaluation of the popliteal hiatus and posterolateral gutter, with dynamic probing of the lateral meniscus and identification MFL tear. The repair is performed through standard anterior portals, using all‐inside vertical sutures to re‐establish continuity between the MFL stump, the inferior border of the posterolateral meniscus, and the posterolateral capsule. This technique allows an anatomic, meniscus‐preserving repair without additional open dissection or hardware in the fibular head. It is reproducible with standard arthroscopic equipment and can be combined with concomitant anterior cruciate ligament reconstruction.

## Graphical abstract

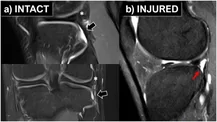

**VIDEO 1:** Arthroscopic diagnosis and all‐inside repair of a meniscofibular ligament tear in a right knee. The patient is positioned supine, and standard anteromedial and anterolateral portals are established. With the knee placed in the figure‐of‐4 position, the lateral compartment is systematically evaluated. Dynamic probing of the posterior horn of the lateral meniscus shows abnormal anterior translation and instability. Inspection of the inferior meniscal surface reveals disruption of the meniscofibular ligament, with visualization of the characteristic “Double Orifice Sign” and increased exposure of the popliteus tendon. The “Probe‐Twirl Test” confirms restoration of physiologic stability when the ligament remnant and inferior meniscal surface are simultaneously engaged. An all‐inside meniscal repair device is introduced through the anteromedial portal, and a vertical mattress stitch is placed to restore continuity between the meniscofibular ligament remnant, the inferior border of the posterior horn of the lateral meniscus, and the posterolateral capsule. After fixation, repeat dynamic assessment shows elimination of abnormal meniscal translation, restoration of the normal relation between the lateral meniscus and the popliteus tendon, and resolution of the pathologic signs of meniscal instability. Video content can be viewed at https://doi.org/10.1002/atn2.70339.

The popliteal hiatus is a complex region where the lateral meniscus loses direct capsular attachment and is stabilized by a network of meniscocapsular and meniscotibial structures, including the 3 popliteomeniscal fascicles (anteroinferior, posterosuperior, and posteroinferior) (PMFs), the lateral and posterior meniscotibial ligaments, the popliteofibular ligament, and the meniscofibular ligament (MFL).^1^, ^2^, ^3^ Together, these structures act synergistically to control the mobility of the posterior horn of the lateral meniscus and maintain stability of the posterolateral compartment.^1^, ^4^

Among these restraints, the MFL represents a distal meniscal attachment extending from the inferior aspect of the posterior third of the lateral meniscus toward the fibular head.^2^, ^5^, ^6^ Anatomical and imaging studies have consistently shown its presence and its continuity with adjacent structures as part of a broader menisco‐tibio‐popliteus‐fibular complex.^2^, ^5^, ^7^, ^8^ By providing an indirect connection between the lateral meniscus, the popliteus tendon, the tibia, and the fibula in an area lacking direct capsular fixation, the MFL plays a complementary role in restraining excessive translation and extrusion of the posterior horn of the lateral meniscus during knee motion, especially during knee extension, varus stress, and external tibial rotation.^1^, ^2^, ^6^ A recent biomechanical study aimed to assess the contribution of posterolateral structures, such as the popliteofibular fascicles, meniscotibial ligaments, and MFL in the lateral meniscus stability, identified the MFL as one of the most important structures. In fact, the selective cut of the MFL was responsible for the largest increase of meniscal anterior displacement, even more than after cutting both the anterior and posterior popliteomeniscal fascicles, thus underlining its central role in meniscal biomechanics.^9^

Given this functional role, the MFL is also exposed to significant tensile and shear forces during the mechanisms typically responsible for anterior cruciate ligament (ACL) injury. Consequently, injury to the MFL and adjacent popliteal hiatus structures may occur concomitantly with ACL rupture, especially in high‐energy pivoting trauma. Indeed, recent clinical studies have reported a substantial incidence of associated posterolateral meniscocapsular injuries in the acute ACL‐injured knee, suggesting that damage to this region is more common than previously recognized.^10^, ^11^

From a clinical standpoint, the diagnosis of MFL injury remains challenging. Symptoms such as posterolateral knee pain, snapping, or mechanical discomfort often overlap with those of ACL deficiency, and specific clinical signs attributable to isolated MFL injury are poorly defined.^5^, ^12^, ^13^ Moreover, standard magnetic resonance imaging (MRI) frequently underestimates or fails to identify these lesions, especially in the acute setting, further contributing to underdiagnosis.^12^, ^14^ As a result, MFL tears may be overlooked during ACL reconstruction, potentially leading to persistent symptoms or lateral meniscal instability if left untreated.

Given these diagnostic limitations, arthroscopy remains the gold standard for the identification of MFL pathology.^1^, ^2^, ^7^, ^15^ However, recognition of MFL tears requires a thorough understanding of the popliteal hiatus anatomy and a systematic, dynamic arthroscopic assessment because these lesions can be subtle and easily missed without targeted inspection. The purpose of this Technical Note is therefore to describe a reproducible, all‐inside arthroscopic technique for the diagnosis and repair of MFL tears both in the setting of ACL injury and in other associated knee pathologies.

## SURGICAL TECHNIQUE

### Step 1: Preoperative MRI Assessment and Indications

Standard radiographs are usually unremarkable and are primarily obtained to exclude osseous abnormalities or associated degenerative changes. MRI plays an important role in the preoperative assessment, mainly to evaluate associated injuries such as ACL tears, posterolateral corner lesions, or chondral damage.^5^, ^13^, ^14^ The MFL can be identified on MRI in more than 40% of cases and typically appears as a low‐signal‐intensity band extending from the inferior aspect of the posterior third of the lateral meniscus toward the fibular head.^5^ Visualization is optimized on thin‐slice and radial sequences. Imaging findings suggestive of MFL or PMF injury include discontinuity, focal thickening with increased signal intensity, or interposed fluid between the lateral meniscus and the fibular head^5^, ^12^ (Figure 1). As a result, imaging alone may underestimate the presence of MFL pathology.^14^ For this reason, definitive diagnosis relies on arthroscopic evaluation, with systematic inspection of the popliteal hiatus, dynamic probing of the posterior horn of the lateral meniscus, and direct visualization of attenuation or disruption of the MFL.^1^

**FIGURE 1:**
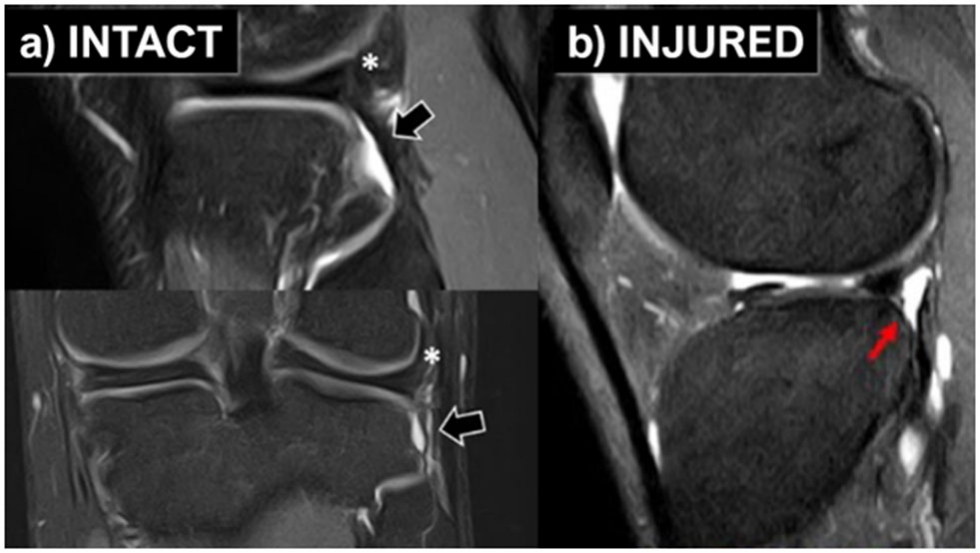
Magnetic resonance imaging evaluation of the MFL in the lateral compartment of the knee. Coronal proton‐density fat‐suppressed MRI of a right knee showing an intact MFL (black arrow) extending from the posteroinferior aspect of the lateral meniscus toward the fibular head and clearly distinct from the popliteus tendon (white asterisk) (a). In contrast, a disrupted MFL is identified as a discontinuity between the posteroinferior margin of the lateral meniscus and the fibular insertion (red arrow) (b), suggesting loss of the normal meniscofibular attachment. (MFL, meniscofibular ligament; MRI, magnetic resonance imaging.)

### Step 2: Patient Positioning and Diagnostic Arthroscopy

Spinal anesthesia is administered. The patient is positioned supine on the operating room table with a foot post and a side support in place. All bony prominences are appropriately padded. A nonsterile tourniquet is applied to the proximal thigh. Intravenous antibiotics are administered less than 30 minutes before incision. A thorough examination under anesthesia is performed to evaluate the degree of laxity as well as to rule out any coronal plane or rotational instability because of collateral ligament injury that should be addressed in surgery. A standard diagnostic arthroscopy is performed using a superomedial portal for water inflow and anteromedial (AM) and anterolateral working portals. The MFL tear is explored and treated before preparation of ACL reconstruction to allow knee manipulation and stress and to avoid bleeding, which could compromise the visualization during the repair technique.

### Step 3: MFL Lesion Arthroscopic Assessment

After inspection of the medial compartment, the lateral compartment is evaluated with the knee placed in the figure‐of‐4 position to allow adequate access to the popliteal hiatus corridor (as shown in Video 1). In a normal knee, the MFL can be appreciated through the standard anterolateral and AM portals. With the probe inserted through the anterolateral portal, the posterior horn of the lateral meniscus and the popliteal hiatus are palpated. Under normal conditions, only a minimal anterior translation (approximately 1‐2 mm) of the posterior horn can be appreciated. By inspecting the inferior meniscal surface, the popliteus tendon can be visualized through the inferior orifice, allowing an indirect assessment of the intact MFL.

By switching the probe to the AM portal, the MFL can be tensioned, and its continuity with the lateral meniscotibial (coronary) ligament can be appreciated. When advancing the arthroscope into the popliteal tunnel, a structure originating from the inferior border of the lateral meniscus and extending distally and laterally can normally be visualized. In the intact condition, this structure partially covers the lateral tibial plateau, and the underlying cartilage is not visible (Figure 2).

**FIGURE 2:**
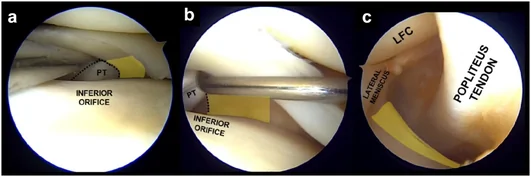
Normal arthroscopic anatomy of the intact MFL in a right knee positioned in the figure‐of‐4 position. The MFL is highlighted in yellow and can be identified through standard anterior portals. (a) The intact inferior orifice viewed from the anterolateral portal, (b) the same structure viewed from the anteromedial portal, and (c) the relation between the MFL and the popliteus tendon from the popliteal tunnel view. In the intact condition, the MFL forms a continuous stabilizing structure along the inferior border of the posterior horn of the lateral meniscus and partially obscures the lateral tibial plateau. (LFC, lateral femoral condyle; MFL, meniscofibular ligament; PT, popliteus tendon.)

In the presence of an MFL injury, the overall meniscal morphology often appears to be preserved, and no gross abnormalities may be evident on routine inspection. However, careful evaluation of the inferior surface reveals a discontinuity between the inferior stabilizing structures. Two distinct openings can be seen: the normal distal inferior orifice and a proximal gap corresponding to the MFL tear. In this setting, a larger portion of the popliteus tendon becomes visible. This is referred to as the “Double Orifice Sign” (Figure 3a).

**FIGURE 3:**
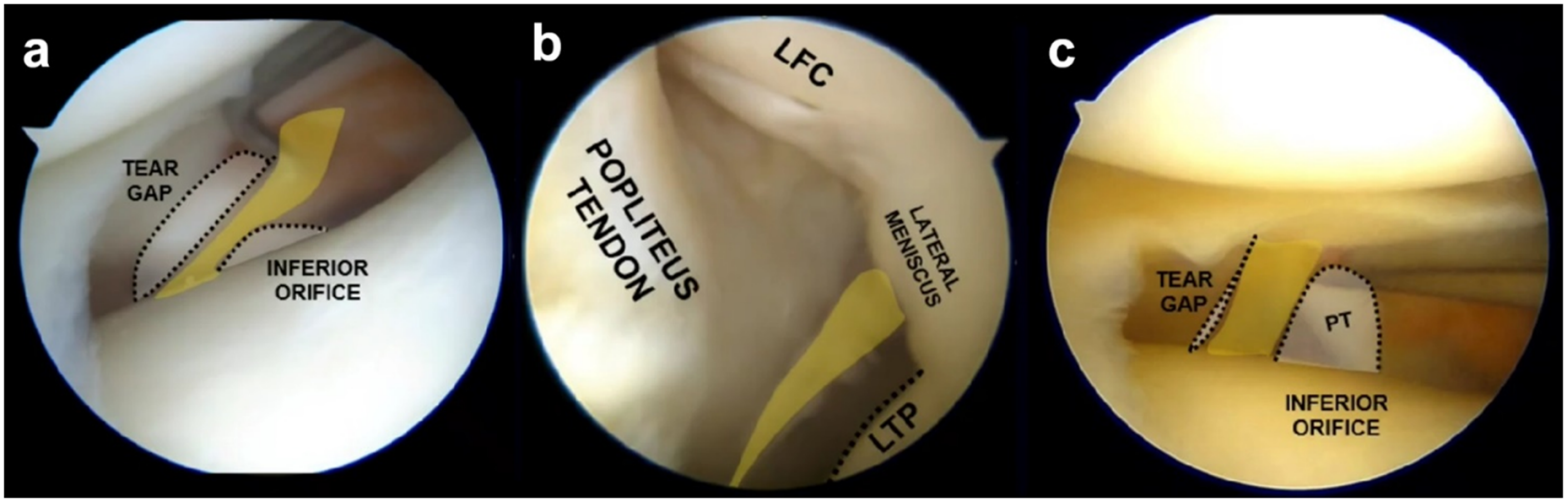
Arthroscopic findings associated with an MFL tear in a right knee positioned in the figure‐of‐4 position. The torn MFL is highlighted in yellow. (a) A tear gap along the inferior surface of the posterior horn of the lateral meniscus, creating the “Double Orifice Sign,” characterized by the presence of both the normal inferior orifice and a second pathologic opening corresponding to the ligament tear. (b) Viewed from the popliteal tunnel, exposure of the lateral tibial plateau cartilage because of loss of the normal meniscofibular restraint, referred to as the “Bare Tibia Sign.” (c) The “Probe‐Twirl Test,” in which the probe simultaneously engages the MFL remnant and the inferior meniscal surface, temporarily restoring ligament tension and physiologic meniscal stability. (LFC, lateral femoral condyle; LTP, lateral tibial plateau; MFL, meniscofibular ligament; PT, popliteus tendon.)

With the arthroscope positioned in the popliteal tunnel, an increased distance between the popliteus tendon and the lateral meniscal wall can be observed, and exposure of the lateral tibial plateau cartilage may be evident; this is referred to as the “Bare Tibia Sign” (Figure 3b). Palpation of the inferior meniscal surface reveals abnormal anterior translation. However, when the probe is inserted beneath the MFL remnant and rotated to engage the inferior meniscal surface, physiologic stability can be temporarily restored. This maneuver represents a positive “Probe‐Twirl test” (Figure 3c).

Furthermore, activation of suction with the shaver results in abnormal anterior displacement and twisting of the lateral meniscus, confirming excessive mobility. This finding represents a positive “Meniscal Suction test.”

### Step 4: MFL Tear Repair Technique

After confirmation of MFL rupture, an arthroscopic all‐inside repair is performed. An all‐inside device is inserted through the AM portal, and the needle is inserted through the inferior meniscal surface proximal to the MFL remnant, followed by the release of the first implant. The needle is removed and inserted distal to the remnant, where the second implant is deployed, creating a vertical mattress stitch configuration. The probe is inserted under the knot while pulling the suture to fine‐tune tension, avoiding overtensioning or iatrogenic injury to the meniscal tissue. The procedure can be performed using commercially available all‐inside meniscal repair devices, according to surgeon preference and local availability. In the present technique, vertical meniscocapsular sutures are placed using an all‐inside meniscal repair system, such as FAST‐FIX 360 or FAST‐FIX FLEX (Smith & Nephew, Andover, MA).

Before cutting the suture, the stability of the repaired meniscofibular complex is carefully reassessed. Adequate restoration of meniscal stability should be confirmed arthroscopically. After final knot advancement and suture cutting, stability is reassessed: the meniscal suction test becomes negative, and advancement of the arthroscope into the popliteal tunnel shows restoration of the normal distance between the popliteus tendon and the lateral meniscal wall. The lateral tibial plateau cartilage is no longer visible. If satisfactory stability is not achieved after the placement of the first stitch, additional vertical mattress sutures may be added. In the present technique, a single well‐positioned stitch was sufficient to restore normal meniscal stability and eliminate the pathologic signs of hypermobility. However, larger lesions or persistent residual laxity may require more than 1 suture, with the final decision based on intraoperative assessment of meniscal stability. Comparison of the pre‐ and postrepair appearance shows closure of the tear gap and restoration of the normal anatomic relation between the meniscus and the posterolateral structures (Figure 4). Technical pearls to facilitate reproducibility of the procedure, along with common pitfalls to avoid, are summarized in Table 1.

**FIGURE 4:**
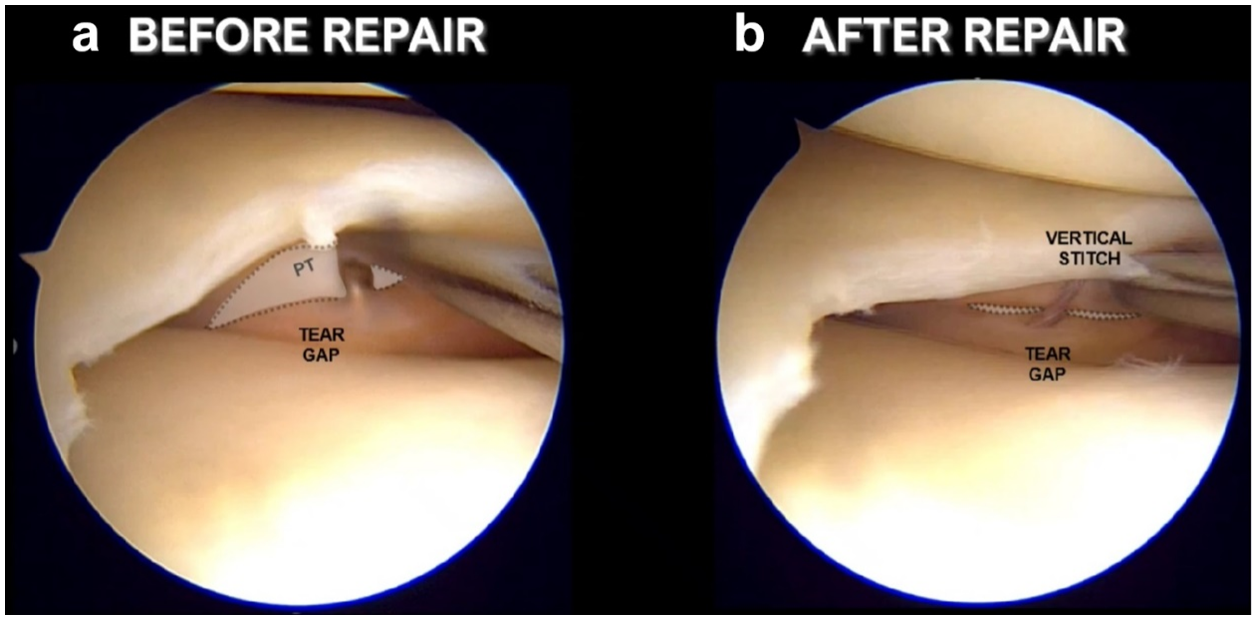
Arthroscopic all‐inside repair of an MFL tear in a right knee positioned in the figure‐of‐4 position. (a) The tear gap between the inferior border of the posterior horn of the lateral meniscus and the MFL remnant before repair. (b) Restoration of continuity of the meniscofibular complex after the placement of a vertical all‐inside mattress stitch, re‐establishing the normal anatomic relation between the lateral meniscus and the posterolateral stabilizing structures. (MFL, meniscofibular ligament; PT, popliteus tendon.)

**TABLE 1:** Pearls and Pitfalls of the All‐Inside Repair of Meniscofibular Ligament

**Pearls**	**Pitfalls**
Perform a systematic evaluation of the posterolateral compartment in all patients with suggestive symptoms	Failure to systematically inspect the popliteal hiatus, leading to missed diagnosis of MFL injury
Use the figure‐of‐4 position to optimize visualization of the popliteal hiatus and lateral meniscus	Misinterpreting normal anatomic variants or synovial folds as pathologic findings
Assess dynamic meniscal behavior with probing to identify abnormal anterior translation or excessive mobility	Relying solely on static inspection without dynamic probing of the lateral meniscus
Look for characteristic findings such as increased distance between the popliteus tendon and lateral meniscus or a double‐orifice sing	Overlooking subtle instability in patients with normal or equivocal MRI findings
Use the “Probe‐Twirl test” and the “Meniscal Suction test” to confirm functional instability	Excessive probing or aggressive suction causing iatrogenic meniscal damage
Clearly identify the MFL remnant prior to suture placement	Inadequate identification of the MFL remnant before suture placement
Place sutures in a vertical mattress configuration to restore continuity between the inferior meniscal border, MFL remnant, and posterolateral capsule	Overtensioning the repair, which may restrict physiologic meniscal motion or cause tissue cut‐through
Adjust suture tension with probe under direct arthroscopic visualization to restore stability while preserving physiologic mobility	Failure to reassess stability after knot tying
Reassess meniscal stability after fixation using dynamic probing and suction testing	Attempting repair in cases with poor tissue quality or advanced degenerative changes
Combine the procedure with ACL or low‐grade posterolateral corner reconstruction when indicated	Ignoring associated ligamentous instability
Select patients with adequate tissue quality to optimize healing potential	Performing the procedure without adequate visualization or improper portal placement

ACL, anterior cruciate ligament; MFL, meniscofibular ligament; MRI, magnetic resonance imaging.

### Postoperative Management

After surgery, an elastic compression bandage is applied and maintained for 12 to 24 hours. Cryotherapy and standard analgesic therapy are administered. No knee brace is used; patients are instructed to keep the lower limb elevated and to maintain the knee in full extension as much as possible during the early postoperative period. Quadriceps setting and isometric strengthening exercises are initiated immediately to preserve muscle activation and minimize atrophy.

Passive range‐of‐motion exercises are started on postoperative day 3 using either a continuous passive motion device or physiotherapist‐assisted manual mobilization, with a gradual increase in flexion to 90° by 15 days postoperatively. Aquatic therapy is permitted 5 to 7 days after suture removal.

In the absence of associated meniscal or osteochondral lesions other than the ACL and MFL, patients are allowed to ambulate with 2 crutches, with a stepwise progression to full weight bearing over 3 to 4 weeks.

Activities requiring deep knee flexion (e.g., squatting) are allowed after approximately 3 months once full range of motion and adequate recovery of muscle tone and trophism have been achieved. Pivoting activities, deep flexion, and torsional loading are restricted during the early rehabilitation phase and are generally permitted starting at 4 months postoperatively. Straight‐line running may be initiated once adequate coordination and neuromuscular control are shown, followed by a progressive return‐to‐running program before sport‐specific drills. Cutting and change‐of‐direction activities are delayed for an additional 2 months.

Return to field‐based athletic activity is allowed when patients are asymptomatic and show symmetric strength and functional performance on clinical testing, most commonly around 5 months postoperatively. Sport‐specific training, including controlled cutting maneuvers and game‐like situations, is typically introduced at approximately 6 months. Return to competitive play is permitted no earlier than 9 months postoperatively, provided that established clinical, functional, and neuromuscular criteria are met.

Final timing is individualized based on intraoperative tissue quality and the stability achieved at the conclusion of the procedure. This rehabilitation strategy aligns with principles used for meniscal‐preserving stabilization procedures and protocols designed to protect meniscocapsular repairs while restoring motion and function.^13^, ^14^

## DISCUSSION

The MFL is an integral component of the posterolateral meniscocapsular complex and contributes to stabilization of the lateral meniscus and the proximal tibiofibular region.^1^, ^3^, ^16^, ^17^ Increasing evidence suggests that injuries to the structures of the menisco‐tibial‐popliteus‐fibular complex commonly occur in the setting of ACL injury, with reported rates reaching up to 20% in ACL‐injured knees,^10^ especially in athletes.^11^ However, despite this association, MFL injury remains largely overlooked in routine clinical practice, and the real incidence is unknown. This is relevant because the MFL has been reported to be responsible of consistent restrain in meniscal anterior displacement,^9^ and thus, the lack of recognizing this injury could theoretically predispose to pain, mechanical symptoms of even meniscal tear progression, as shown for the medial meniscus in the case of unrecognized ramp tears.^18^ Other studies investigating untreated posterolateral meniscocapsular injuries have shown associations with persistent mechanical symptoms, lateral meniscal hypermobility, and progressive cartilage degeneration of the lateral compartment.^14^, ^19^ Notably, Guimarães et al. showed that PMF tears in ACL‐injured knees are associated with accelerated lateral femoral cartilage degeneration over a 2‐year period, underscoring how residual posterolateral instability may compromise long‐term joint health despite restoration of ACL stability.^8^

Diagnosis of MFL injury remains one of the main challenges.^12^ These lesions are frequently occult on MRI and may contribute to persistent symptoms or failure after ligament reconstruction when left untreated.^1^, ^8^, ^19^ Arthroscopic identification of the MFL requires familiarity with the anatomy of the posterolateral gutter and a dynamic evaluation of which is not routinely incorporated into standard diagnostic arthroscopy.

The technique described in this Technical Note emphasizes a systematic arthroscopic assessment of the MFL. The described signs (“Double Orifice” and “Bare Tibia”) and tests (“Meniscal Twirl” and “Meniscal Suction”), despite not being formally validated, offer an important aid to clinicians to assess—or at least consider—lateral meniscus laxity. It is suggested to perform this assessment in every arthroscopic case to develop familiarity with normal and pathological anatomy.

Regarding the surgical technique, the described all‐inside repair restores continuity between the inferior border of the lateral meniscus, the MFL remnant, and the posterolateral capsule, re‐establishing physiologic restraint of the posterior horn. Importantly, this technique can be performed using standard arthroscopic portals and instrumentation and can be readily combined with ACL reconstruction without the need for accessory portals or specialized equipment. This allows an easy and rapid treatment of a frequently neglected lesion within the same surgical setting in which ACL stability is restored without adding significant operative time or morbidity. The technique presented builds on previously described strategies for treating hypermobile lateral meniscus caused by PMF tears or insufficiency.^13^, ^14^, ^20^ Prior clinical series have shown that arthroscopic stabilization of the posterior horn using all‐inside or suture‐based techniques leads to reliable improvement in pain and mechanical symptoms, with low revision rates.^14^ Our approach extends these principles to MFL lesions.

The proposed technique offers several advantages. First, it allows direct arthroscopic diagnosis and treatment of a frequently overlooked lesion without the need for additional incisions or fixation at the fibular head. The procedure can be performed through standard anterior portals using commonly available all‐inside meniscal repair devices, making it easily reproducible for surgeons familiar with arthroscopic meniscal repair. Furthermore, preservation of the native MFL remnant and restoration of the physiologic relation between the lateral meniscus and the posterolateral structures may help maintain normal lateral compartment biomechanics while avoiding excessive constraint of the posterior horn.

Several limitations should be acknowledged (the advantages and limitations of the technique are reported in Table 2). Diagnosis remains highly dependent on arthroscopic expertise and familiarity with the anatomy of the popliteal hiatus. Furthermore, the proposed arthroscopic signs and dynamic tests have not yet been formally validated, and their diagnostic accuracy remains unknown. The optimal number and configuration of sutures have also not been established; although a single vertical mattress stitch was sufficient in our experience, larger lesions or persistent residual laxity may require additional fixation. As with other lateral meniscal stabilization procedures, excessive tensioning may result in overconstraint and alteration of the physiologic mobility of the posterior horn. The described technique also requires adequate tissue quality and may be more challenging in chronic cases with advanced degenerative changes. Finally, clinical outcomes and healing rates after isolated MFL repair remain unknown. Although early clinical experience supports the value of concomitant repair of posterolateral meniscal stabilizers, larger case series and prospective studies are needed to better define indications, validate diagnostic criteria, and quantify the long‐term impact of MFL repair on meniscal stability and lateral compartment preservation.

**TABLE 2:** Advantages and Disadvantages of Arthroscopic All‐Inside Meniscofibular Ligament Repair

**Advantages**	**Disadvantages**
Fully arthroscopic procedure without additional incisions	Requires familiarity with popliteal hiatus anatomy
Simultaneous diagnosis and treatment of MFL tears	Arthroscopic signs and tests are not yet validated
Uses standard anterior portals	Diagnosis remains operator‐dependent
Uses commonly available all‐inside repair devices	Limited evidence regarding clinical outcomes
Preserves native meniscal tissue and ligament remnant	Optimal number and configuration of sutures remain unknown
Easily combined with ACL reconstruction	Risk of insufficient stabilization if lesion extent is underestimated
No hardware placement at the fibular head	May not be suitable in severely degenerative tissue
Allows intraoperative dynamic assessment of repair stability	Potential risk of overconstraint if excessive tension is applied

ACL, anterior cruciate ligament; MFL, meniscofibular ligament.

## DECLARATION OF GENERATIVE AI AND AI‐ASSISTED TECHNOLOGIES IN THE WRITING PROCESS

During the preparation of this work, the authors used ChatGPT, OpenAI to assist with language editing. After using this tool/service, the authors reviewed and edited the content as needed and take full responsibility for the content of the publication.

## DISCLOSURES

The authors (A.G., T.C., V.G.R., C.R., G.M.M.M., S.Z.) declare that they have no known competing financial interests or personal relationships that could have appeared to influence the work reported in this article.

## FUNDING

Open access publishing facilitated by Universita di Bologna, as part of the Wiley ‐ CRUI‐CARE agreement.
